# Impact of mini-implant length on stability at the initial healing period: a controlled clinical study

**DOI:** 10.1186/1746-160X-9-30

**Published:** 2013-10-20

**Authors:** Manuel Nienkemper, Benedict Wilmes, Alexander Pauls, Dieter Drescher

**Affiliations:** 1 Department of Orthodontics, Heinrich-Heine-University, Moorenstr. 5, 40225 Düsseldorf, Germany; 2 Orthodontist, private practice, Gersthofer Str. 2, 65929 Frankfurt am Main, Germany

**Keywords:** Skeletal anchorage, Mini-implants, Stability, Length, Healing, Resonance frequency analysis

## Abstract

**Introduction:**

Aim of the study was to assess the impact of the length of mini-implants inserted in the midpalatal region on the stability at the initial healing period.

**Methods:**

A sample of 20 consecutively treated patients (15.6 ± 7.2 years) was examined. A long mini-implant with a length of 11 mm and a diameter of 2 mm was inserted into the anterior palate of each patient. Resonance frequency analysis (RFA) was performed after insertion (T0), two weeks (T1), four weeks (T2), and six weeks (T3). Insertion depth (ID) and the maximum insertion torque (IT) were measured. RFA, ID and IT data were tested for correlations. RFA values were tested for statistical differences between the different times. Data was compared to a matched control group of patients who received short mini-implants with a length of 9 mm and a diameter of 2 mm.

**Results:**

Mean ID was 9.5 ± 0.6 mm and mean IT was 17.9 ± 3.8 Ncm. A correlation was found between RFA and ID (r = 0.59, *P* < .01). From T0 to T1 the stability (33.4 ± 3.5 ISQ) decreased highly significantly by 5.3 ± 3.5 ISQ values (*P <* .001) and significantly from T1 and T2 (*P* < .05) by 3.5 ± 3.7 ISQ values. From T2 on RFA nearly remained unchanged (−1.7 ± 3.9 ISQ; *P* > .05). At T1 stability was significantly lower than the control group. From T2 on there were no significant differences between the groups.

**Conclusions:**

Long mini-implants provide high stability when inserted in the midpalatal region. After initial decrease RFA values remained stable from four weeks on and did not differ from the control group.

**Trial registration:**

ID: 2013081293 (Clinical study register, University of Düsseldorf, Germany).

## Introduction

Anchorage control is a key factor for successful treatment in orthodontics. Skeletal anchorage can be very helpful especially in critical anchorage situations. Dental implants [1], palatal implants [2] or mini-plates [3] were used for this purpose. Mini-implants, introduced by Kanomi, have become increasingly widespread in the recent past because of their low invasiveness during insertion and removal, their versatility and low costs [4]. However, relatively high failure rates ranging from 10% up to 30% remain the major problem using this type of temporary anchorage devices [5]. For that reason researchers have been working on various designs in order to optimize mini-implant stability:

There are some studies assessing the influence of different materials [6] or surface treatment [7] to enhance cellular reaction and micro retention. Other studies focus on macroscopic design elements such as thread and taper design [8]. Different lengths and diameters as well as conical or cylindric shapes were tested for their influence on primary stability [9,10]. Most of these investigations are *in-vitro* studies. In the clinical studies insertion torque is mostly used as a measure for primary stability. The validity of this parameter to assess implant stability and to predict success, however, is still controversial [11]. In addition it only allows assessing initial stability but not secondary stability which is even of more relevance for clinical success.

Up to now, all the studies investigating the influence of different mini-implant designs are confined to primary stability measurement. Without doubt, primary stability is a basic prerequisite for clinical success [12]. But due to the remodelling process that occurs at the bone to implant interface during the healing period, stability is significantly affected [13]. Hence, it would be highly interesting to investigate the influence of different design features on primary *and* secondary stability of orthodontic mini-implants [14,15]. In dental implantology resonance frequency analysis (RFA) is established for clinical stability measurement subsequent to insertion and at any time during further treatment [16,17]. In the recent past RFA has proven to be suitable also for assessment of mini-implant stability [18].

The aim of this clinical study was to determine the stability of long mini-implants with a length of 11 mm at the initial healing period of six weeks and to compare it with the results of shorter mini-implants with a length of 9 mm derived from a previous pilot study [19].

## Materials and methods

### Subjects

Only patients whose treatment plan comprised the insertion of palatal mini-implants were taken into consideration. Absence of systemic diseases affecting bone metabolism or wound healing and good oral hygiene were further inclusion criteria. Patients who missed examination appointments or showed signs of peri-implant inflammation were excluded. Participation was non-mandatory, each patient signed a patient consent form.

### Power analysis

For sample size calculation data of a clinical pilot study was used. This study investigated the stability at the initial healing period of palatally inserted mini-implants with a length of 9 mm, based on the same observation protocol. Significant changes in stability were found between the second and the fourth week. Consequently, ISQ changes and standard deviations of this observation period were used for calculation.

The changes in ISQ values of 7.89 with a standard deviation of 5.92, a chosen alpha level of 0.001 and a power of 0.95 resulted in a required sample size for the treated and the control group of n = 19. G*Power 3.1.5 (University of Kiel, Germany) software was chosen for this calculation. During the pilot study 4 patients had to be excluded either due to signs of peri-implant inflammation or missed examination appointments. As a consequence 23 consecutively treated patients were examined for this study. No further selection was performed.

Having 3 drop outs the test group finally comprised 20 patients, 10 males and 10 females of white ancestry with a mean age of 15.6 ± 7.2 years (Table 1).

**Table 1:** Comparison regarding gender and age between the groups

	**2 × 11 mm**		**2 × 9 mm**			
Gender:	10 male	10 female	11 male	8 female	n.s.	Chi-square-test
Age (years):	15.62	7.19	15.55	7.34	n.s.	Mann.Whitney-*U*-Test
* p < .05	**p < .001	***p < .0001				

The prospective study was approved by the ethical committee of the University Clinics of Düsseldorf, Germany. It was performed according to the Declaration of Helsinki guidelines on experimentation involving human subjects.

### Insertion protocol

**Figure 1:**
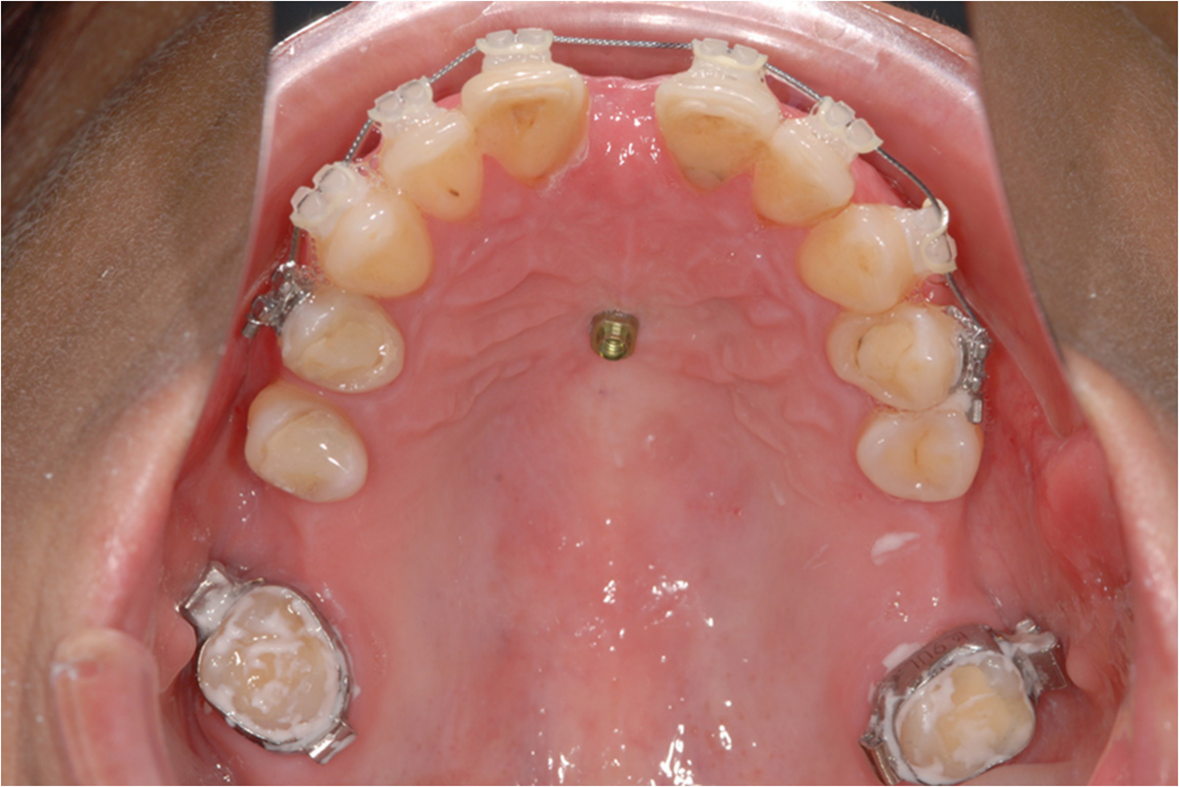
2 × 11 mm mini-implant inserted in the median part of the anterior palate between the second and third palatine rugae.

Benefit mini-implants (length: 11 mm, outer diameter: 2 mm, inner diameter: 1.35 mm, thread pitch: 0.75 mm) (PSM medical solutions; Tuttlingen, Germany) were inserted in the midpalatal suture between the second and third palatine rugae (Figure 1). After predrilling using a 1.3 mm drill to a depth of 3 mm insertion was performed rectangular to the palatal curvature until the mini-implant’s head touched the soft tissue. A surgical machine (ElcoMed SA 200C, W&H, Bürmoos, Austria) was used for predrilling and insertion.

### Observation protocol

Prior to insertion the soft tissue thickness (STT) was determined using a dental probe with a rubber stop. Insertion depth (ID) was calculated subtracting these values from the mini-implants’ thread length (11 mm). During insertion the maximum insertion torques value (IT) were recorded by the surgical machine. After insertion the RFA was performed three times along the midpalatal suture and three times perpendicular to it (T0). RFA measurements were repeated after 2 (T1), 4 (T2) and 6 (T3) weeks. Mean ISQ values were calculated from the repeated measurements in both directions and as overall values.

Peri-implant soft tissue was each time observed for signs of inflammation.

### Matching of the control group

The data was additionally compared to those of the already mentioned pilot-study. In that study the same observation protocol was used to evaluate the changes in stability of 2 × 9 mm Benefit mini-implants. The control group consists of 19 patients, 11 males and 8 females of white ancestry with a mean age of 15.5 ± 7.3 years. The control group matched the treatment group as to sex distribution and age (Table 1).

The only differences between the two groups were the length of the mini-implants and the spot for insertion, which is a little bit more anterior in this study.

### Statistical analysis

Normal distribution (Shapiro-Wilk test) as well as equality of variances (Levene-test) were found for all ISQ and ID values. Only IT values did not show normal distribution. Hence ANOVA and Duncan post hoc test as parametric tests were applied to assess differences between the ISQ values at T0, T1, T2 and T3. For comparison of ISQ values parallel and perpendicular to the midpalatal suture the paired *t*-test was used. Pearson correlation and linear regression analysis were chosen to analyze relations between initial ISQ, IT, and ID values.

For inter-group comparison unpaired *t*-test was used for differences in ISQ values at each observation appointment and in ID values. For comparison of IT values Mann–Whitney-*U*-test was chosen since the respective data did not show normal distribution for the 2 × 11 mm mini-implants.

Statistics were performed with the statistical software SPSS 21.0 (IBM, Chicago, Ill). Statistical significances were tested at *P* < .05 (*), *P* < .001 (**) and *P* < .0001 (***) levels.

## Results

Soft tissue thicknesses at the insertion site were found to be nearly identical in both groups (STT: 1.55 ± 0.60 mm vs. 1.50 ± 0.55 mm). Given the higher length of the implants used in the present study the insert depth was nearly 2 mm higher than in the pilot study (ID: 9.45 ± 0.60 vs. 7.50 ± 0.55) (Table 2).

**Table 2:** Initial values for RFA, insertion torque (IT) and soft tissue thickness (ST)

	**2 × 11 mm**		**2 × 9 mm**			
	**mean**	**SD**	**mean**	**SD**	**p**	
STT (mm)	1.55	0.6	1.5	0.55	n.s.	*t*-test
ID (mm)	9.45	0.60	7.5	0.55	***	*t*-test
IT (Nmm)	17.85	3.75	16.81	3.54	n.s.	*U*-test
RFA (ISQ)	33.35	3.53	36.14	6.08	n.s.	*t*-test
* p < .05	**p < .001	***p < .0001				

Mean IT was 17.85 Ncm and mean initial ISQ was 33.35 ± 3.53. In relation to the control group IT was slightly higher whereas RFA showed lower values. Differences did not reach the level of significance.

Within the test group a significant correlation could be found between ID and initial ISQ values with a correlation coefficient of r = 0.59 (Table 3). Between ID and IT and between RFA and IT there was no correlation.

**Table 3:** Correlations between RFA, insertion torque (IT) and soft tissue thickness (ST) (2 × 11 mm)

	**r**	**p**	**r**^**2**^
ID - IT	0.31	0.18	0.1
RFA-IT	0.26	0.27	0.07
RFA-ID	0.59	<0.01	0.35

**Figure 2:**
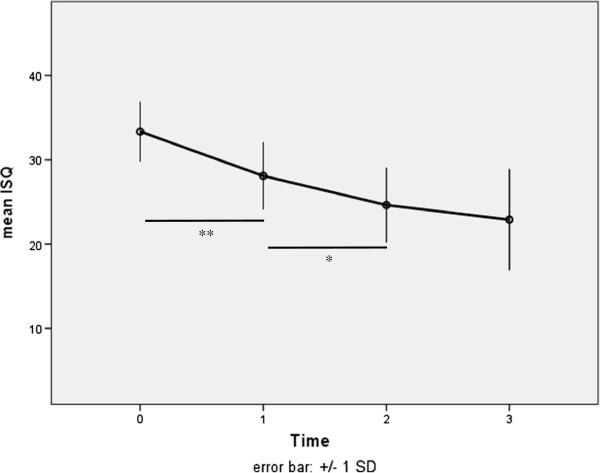
Stability of 2 × 11 mm mini-implants over six weeks by means of RFA (ISQ).

The stability shown by RFA measurements was subject to changes over the first six weeks. The initial ISQ of 33.35 ± 3.53 decreased significantly during the first two weeks by 5.25 ± 3.53 (p < .001) and during week three and four by 3.45 ± 3.66 (p < .05) (Tables 4 and 5); Figure 2. From week four on the stability remained nearly unchanged. Overall the stability decreased significantly by 10.45 ± 5.22 ISQ to a mean level of 22.9 ± 6.0. There were no statistical differences regarding measurement direction.

**Table 4:** RFA values (ISQ) at each measuring point (2 × 11 mm)

	**T0**		**T1**		**T2**		**T3**	
	**mean**	**SD**	**mean**	**SD**	**mean**	**SD**	**mean**	**SD**
Overall	**33.35**	3.53	**28.10**	3.99	**24.63**	4.46	**22.90**	6.00
length	33.55	3.92	28.35	4.43	24.62	4.39	23.30	5.90
square	33.15	3.27	27.85	3.91	24.65	4.82	22.50	6.30
	ns		ns		ns		ns	
* p < .05	**p < .001	***p < .0001						

**Table 5:** Changes in mini-implant stability over time measured by RFA (ISQ)

	**T1-T0**		**T2-T1**		**T3-T2**		**T3-T0**	
	**mean**	**SD**	**mean**	**SD**	**mean**	**SD**	**mean**	**SD**
Test (2x11mm)	−5.25	3.53	−3.45	3.66	−1.73	3.88	−10.45	5.22
	**		*		ns		***	
Control (2x9mm)	−4.03	6.08	−7.89	5.92	−1.72	3.53	−13.63	9.49
	ns		**		ns		***	
	* p < .05	**p < .001	***p < .0001					

**Figure 3:**
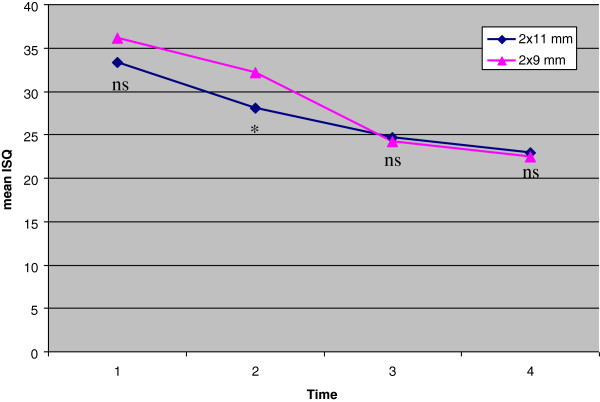
Comparison of the stability of 2 × 11 mm and 2 × 9 mm mini-implants over six weeks by means of RFA (ISQ).

The comparison with the control group showed significant lower stability at T1 after comparable initial ISQ values due to the significant decrease of stability from T0 to T1 in the test group (p < .001) (Table 6, Figure 3). From T1 to T2 there is a slight decrease (p < .05) whereas ISQ values in the control group decrease by 7.89 ± 5.92 (p < .001). As a result the stability at T2 is nearly the same in both groups. From T2 to T3 stability remains nearly unchanged in both groups.

**Table 6:** Comparison of RFA values (ISQ) at each measuring point

	**T0**		**T1**		**T2**		**T3**	
	**mean**	**SD**	**mean**	**SD**	**mean**	**SD**	**mean**	**SD**
Test (2x11 mm)	**33.35**	3.53	**28.1**	3.99	**24.63**	4.46	**22.9**	6
Control (2x9 mm)	**36.14**	6.08	**32.11**	5.57	**24.23**	7.19	**22.51**	6.69
Difference	**−2.79**		**−4.01**		**0.4**		**0.39**	
p	0.086		0.013		0.833		0.848	
	n.s.		*		n.s.		n.s.	
* p < .05	**p < .001	***p < .0001						

## Discussion

For this prospective clinical study a sufficient number of patients could be included after drop outs, as calculated by power analysis on the basis of a clinical investigation using a nearly identical procedure [19]. The patients of that pilot study were found to be suitable as control group to identify the effects of implant length on the primary and secondary stability, since they match the test group as to age and gender. A test for normal distribution for small sample sizes was used to select appropriate statistics. The measurement methods used for stability assessment have proven to be accurate enough for this purpose [18,20].

The results indicated a high primary stability as shown by the RFA and IT values at T0. Interestingly, the stability was not higher than those of the 9 mm long mini-implants of the control group. In many studies stability is determined by measurement of maximum insertion torque [14,15].

A closer look into the literature addressing the impact of implant length reveals that different mesurement methods and implant loading modes may characterize different aspects of implant stability:

Pithon et al. reported higher maximum insertion torque values, when longer mini-implants were used [21]. The current results show the same tendency, but yet not significantly. But in that *in vitro* study higher implant length did not lead to higher mechanical resistance when loaded by a lateral force. Chatzigianni et al. found that the mini-implant’s length only affected lateral dispalcement at a particular force value [22]. In our study the ISQ values of the longer mini-implants were lower than those of the control group but without reaching the level of significance. Pan et al. reported of lower ISQ values for longer mini-implants and stated that insertion depth related to the absolute implant length is of higher importance for stability and mechanical load capacity. The current investigation also found a corraltion between RFA and ID whereas no correlation was found between RFA or ID to IT. The fact that orthodontic mini-implants are primarily loaded by lateral forces suggests that RFA might be more suitable to detect this kind of stability than IT.

The direction of RFA measurement after insertion at the midpalatal region showed no significant differences as it was already presumed by the results of the pilot-study.

The position of the mini-implants might have a minor impact on stability. The longer mini-implants were inserted slightly more anteriorly than in the control group (directly distal from the third palatine rugae) due to greater bone height in this area [23]. They consequently came closer to the incisive canal. Song et al. investigated the microanatomy of the incisive canal and found that there is a high variation in shape, diameter and course. Even the number of canals ranged from one to four. As a result, no guidelines can be given to the clinician how to reliably prevent touching or penetrating the canal. But the higher length, the position and axis of the mini-implants superimposed with the anticipated path of the incisive canal give reason to presume that the canal was hit in some cases. Somehow, this might affect mini-implant stability. The authors also found that there were numerous arteries and veins as well as bundles of small nerves in relatively large canals. Since there is plenty of space within the canal, the small nerve fibres may easily slip away when a mini-implant penetrates. Hence, sequelae such as numbness of the anterior palatal mucosa due to damage of the nasopalatine nerve are rather unlikely and were not observed, yet. Also in the present and the pilot study none of the patients complained about numbness at the respective soft tissues.

The current investigation suggests that longer, i.e. 11 mm mini-implants in the anterior palate that have to be inserted slightly more anteriorly due to bone height do not provide higher stability than 9 mm mini-implants, neither immediately after insertion nor after the initial healing period of six weeks. After two weeks stability was even lower. This is due to a significant decrease (−5.25 ± 3.53 ISQ; p < .001) from T0 to T1 whereas the shorter implants of the control group showed no significant stability changes. During T1 and T2 when the control group had the major loss of stability (−7.89 ± 5.92 ISQ; p < .001) the decrease of the 2 × 11 mm mini-implants flattened, but was still significant (3.45 ± 3.66; p < .05). From week four on the stability remained constant in both groups showing no significant differences.

Overall the development of stability was similar in both groups. Stability decreased significantly for the first four weeks in accordance to dental implants that showed lowest stability after three [24] or four [25] weeks. Stability of surface treated implants already had begun to increase after six weeks [26] whereas mini-implant’s stability of both groups remained unchanged. For orthodontic purposes this level of stability seems to be suitable. It also should be kept in mind that excessively high stability levels may be detrimental, since temporary anchorage devices of small diameter might break at the time of removal [27].

By now, there is no study evaluating the influence of mini-implant length on secondary stability. Sim et al. investigated the development of stability of dental implants 8 mm and 10 mm in length [28]. Over the first two weeks initial differences in stability were found that did not reach the level of significance due to high standard deviations. After that stability developed nearly identically independent from implant length.

Further clinical investigations regarding the factors that may affect primary and secondary stability such as different mini-implant diameters, surface treatment or different insertion sites should be performed to optimize the clinical protocols and success rates of skeletal anchorage.

## Conclusions

Long 11 mm mini-implants provide a high level of stability when inserted at the midpalatal region.

After initial loss the stability did not change significantly from week four on. After the first two weeks the longer mini-implants inserted in a more anterior position even showed a lower stability than 9 mm long mini-implants. After four weeks the stability was comparable. Regarding stability there seems to be no advantage in using longer mini-implants at the median part of the palate. Preferring short implants of 9 mm length inserted slighty posteriorly may also help to prevent hitting the incisive canal even though this might not be of clinical relevance.

## Abbreviations

RFA: Resonance frequency analysis; ISQ: Implant stability quotient; IT: Maximum insertion torque value; ID: Insertion depth; STT: Soft tissue thickness.

## Competing interests

Dr. Wilmes is the inventor of the Benefit System.

## Authors’ contributions

MN performed the measurements, statistical analysis and drafted the manuscript. AP coordinated patients’ appointments and took part in measuring. DD and BW took part in writing the paper. All authors read and approved the final manuscript.
